# Case of Idiopathic Hydrophobia

**Published:** 1843-03

**Authors:** J. Kimbell


*Case of Idiopathic Hydrophobia.*—By J. Kimbell, M. R. C. S. L.—W. K., aged twenty-four years, of a bilio-lymphatic temperament, has, during the last month, suffered from occasional attacks of palpitation of the heart, occurring generally in the night, and invariably followed by profuse perspiration. On October 4th, 1841, he rode a distance af fourteen miles, and on arriving at the end of his journey at about twelve o'clock, A. M., he was seized suddenly with great difficulty of breathing, pain over the region of the heart, and painful sensations over the chest. The paroxysm continued for a few minutes, when the dyspnœa and pain gradually subsided; he afterwards ate a good dinner, and appeared as well as usual, until about eight o'clock in the evening. when all the symptoms returned with greater violence than before, and to so distressing a degree did the dyspnœa increase, that there appeared to be imminent danger of suffocation. He was now bled to eighteen ounces, but without any manifest relief, and the operation was repeated in three hours to the amount of six ounces, which had the effect of considerably relieving the pain.

About five, A. M., October 5th, I saw him; he could not speak, although conscious of what was passing around him; I was informed that he had had violent convulsive movements of the arms, which had lasted nearly an hour, and he now appeared to be suffering from a spasmodic constriction about the glottis and pharynx, causing extreme difficulty of inspiration, which had a peculiar crowing character; he had likewise a great desire for water, and complained much of thirst. No sooner, however, was this fluid brought into his presence than it was obliged to be withdrawn; the sight of it caused an alarming increase of pain about the larynx, with a horrible feeling of suffocation; but with the removal of the water the symptoms became ameliorated. From so many hydrophobic symptoms being present, I was apprehensive he might have been bitten by a dog, so questioned him upon this subject very closely; but to all my interrogations he shook his head negatively. During the intervals of ease his pulse was full and soft, and averaged eighty beats in a minute; his tongue was clean, the bowels were regular, and the skin of the natural temperature. Aware that there was a predisposition to spinal disease, I examined the back, and found about the lower part of the cervical region tenderness on pressure, and I observed that this pressure invariably produced an exacerbation in all the symptoms, and of this I fully satisfied myself, and my patient likewise, by repeating the pressure three or four times. A blister was applied over this spot; it rose well, and he soon became able to swallow. Doses of opium were given by the mouth, and an opium injection was administered per rectum. I should have stated that from the commencement of the attack up to the present period, he has experienced a great difficulty in passing his urine, but none in voiding his fæces.

5. Much improved in every respect; but when his head was raised, the spasm was speedily reproduced. He had a constant smacking of his lips, and frequent twitches of his legs and feet; the right arm partially paralysed; no headache; no confusion of intellect.

7. Still improving; spasms had entirely disappeared; he could swallow fluids with the greatest ease; tongue clean; bowels well opened; secretions healthy; he can now be raised without suffering; the blister discharges freely. The dorsal region was rubbed with an embrocation, containing croton oil, tartar emetic, &c., and quinine was given during the day, with henbane at night. From this period he gradually progressed, and at the end of the month was thought sufficiently improved to resume his avocation. One day, however, previous to his intended departure, he had a recurrence of the dyspnœa, but in a much less degree than before. This was immediately treated by the application of leeches to the cervical region, followed by a blister, when all the symptoms soon vanished. He has two issues, one on each side of the cervical vertebræ, which discharge freely, and he may now be considered convalescent.—*London Lancet.*

